# Importance of Thyroid Hormone level and Genetic Variations in Deiodinases for Patients after Acute Myocardial Infarction: A Longitudinal Observational Study

**DOI:** 10.1038/s41598-020-66006-9

**Published:** 2020-06-08

**Authors:** Nijole Kazukauskiene, Daina Skiriute, Olivija Gustiene, Julius Burkauskas, Violeta Zaliunaite, Narseta Mickuviene, Julija Brozaitiene

**Affiliations:** 1 Laboratory of Behavioral Medicine, Neuroscience Institute, Lithuanian University of Health Sciences, Vyduno al. 4, LT-00135 Palanga, Lithuania; 2 Laboratory of Molecular Neurooncology, Neuroscience Institute, Lithuanian University of Health Sciences, Eiveniu str. 4, LT-50161 Kaunas, Lithuania; 3 Department of Cardiology, Medical Academy, Lithuanian University of Health Sciences, Eiveniu str. 2, LT-50009 Kaunas, Lithuania; 4 Laboratory of Behavioral Medicine, Neuroscience Institute, Lithuanian University of Health Science, Vyduno al. 4, LT-00135 Palanga, Lithuania; 5 Laboratory of Behavioral Medicine, Neuroscience Institute, Lithuanian University of Health Science, Lithuanian University of Health Sciences, Eiveniu str. 2, LT-50009 Kaunas, Lithuania; 6 Laboratory of Behavioral Medicine, Neuroscience Institute, Lithuanian University of Health Science, Lithuanian University of Health Sciences, Vyduno al. 4, LT-00135 Palanga, Lithuania

**Keywords:** Cardiology, Cardiovascular biology, Genetics, Clinical genetics

## Abstract

This study aimed to examine the influence of thyroid hormone (TH) levels and genetic polymorphisms of deiodinases on long-term outcomes after acute myocardial infarction (AMI). In total, 290 patients who have experienced AMI were evaluated for demographic, clinical characteristics, risk factors, TH and NT-pro-BNP. Polymorphisms of TH related genes were included deiodinase 1 (*DIO1*) (rs11206244-C/T, rs12095080-A/G, rs2235544-A/C), deiodinase 2 (*DIO2*) (rs225015-G/A, rs225014-T/C) and deiodinase 3 (*DIO3*) (rs945006-T/G). Both all-cause and cardiac mortality was considered key outcomes. Cox regression model showed that NT-pro-BNP (HR = 2.11; 95% CI = 1.18– 3.78; p = 0.012), the first quartile of fT3, and *DIO1* gene rs12095080 were independent predictors of cardiac-related mortality (HR = 1.74; 95% CI = 1.04–2.91; p = 0.034). The *DIO1* gene rs12095080 AG genotype (OR = 3.97; 95% CI = 1.45–10.89; p = 0.005) increased the risk for cardiac mortality. Lower fT3 levels and the *DIO1* gene rs12095080 are both associated with cardiac-related mortality after AMI.

## Introduction

Recent clinical research in cardiovascular disease as well as in coronary artery disease (CAD) has provided evidence that altered thyroid hormone (TH) metabolism, including low total triiodothyronine (T3) syndrome or pre-existing subclinical primary hypothyroidism, is an important indicator of adverse short-term and long-term outcomes, including mortality^1–5^. These changes in thyroid homeostasis are known as “euthyroid sick syndrome”^6,7^ or “non-thyroidal illness syndrome” (NTIS)^8,9^ and are defined by low serum levels of free T3 (fT3), T3 and high levels of reverse T3 (rT3) followed by normal or low levels of thyroxine (T4) and thyroid-stimulating hormone (TSH). Low T3 syndrome is observed in about one third of patients following acute cardiovascular events and has been linked to the severity of the disease and its adverse prognosis^10^. This syndrome has been established in patients with heart failure (HF)^11–14^, myocardial infarction (MI)^2,15–18^, and has been linked to the cardiac remodelling process^19–21^ and poor prognosis^1,3,4,13,22,23^. Studies suggest that variations of TH within clinically normal ranges, such as isolated reduction in fT3 level or higher level of free T4 (fT4), could constitute a model of abnormal TH metabolism. These variations could act as a risk factor for CAD, in a similar fashion to overt or subclinical hypothyroidism, thereby influencing the occurrence as well as severity of coronary atherosclerosis and its related outcomes^2,24–32^.

Recent studies acknowledge the influence of common genetic variations in TH pathway genes on thyroid function^33–36^. The production of TH, in particular the prohormone T4, is controlled by the hypothalamic-pituitary-thyroid axis, whereas its biological activity is primarily regulated by iodothyronine deiodinases enzymes. Maintenance of euthyroidism at a serum level and peripherally is determined by deiodinase type 1 (*DIO1*), deiodinase type 2 (*DIO2*) and deiodinase type 3 (*DIO3*)^37–41^. Both *DIO1* and *DIO2* are predominantly activating enzymes and convert T4 to T3 and rT3 to diiodothyronine (T2), while *DIO3* inactivates TH and converts T3 to T2 and T4 to rT3^20,37,38,42,43^. During recent years, it has been demonstrated that certain genetic polymorphisms in gene coding for deiodinases could alter gene function and are associated with variations in TH levels, such as fT3, fT4, T4 and rT3 in hypothyroid patients, healthy individuals^34,42,44,45^ and CAD patients^46^.

To our knowledge, there are no reports studying the association between circulating TH ranges and genetic variability of genes related to TH axis on the long-term mortality in CAD patients after acute MI (AMI). Our study aimed to examine the prognostic importance of TH level and genetic polymorphisms *DIO1, DIO2*, and *DIO3* on long-term outcomes in patients with CAD after AMI.

## Methods

### Study population

In total, 330 AMI patients with ST-segment elevation and non ST-segment elevation in the cardiac Intensive Care Unit (ICU) at the Lithuanian University of Health Sciences Hospital were invited to participate in the study. Standard treatment had been given according to the existing guidelines for AMI management^47–50^. Inclusion criteria covered ages over 18 years and an AMI diagnosis. Patients were excluded if they were taking thyroid medications or amiodarone, had increased levels of TSH ( > 4.8 mIU/l), indicating hypothyroidism, reduced TSH ( < 0.5 mIU/l), indicating hyperthyroidism, or if they had serious systemic disease (e.g. cancer, autoimmune disease, or chronic renal disease). All eligible participants provided written informed consent. The final study population was comprised of 290 patients with AMI (72% men and 28% women; mean age, 62 ± 11 years).

### Study design

Eligible participants were evaluated for socio-demographic factors and clinical characteristics such as history and type of AMI, HF, left ventricular ejection fraction (LVEF), Killip class, and current medication use. Participants were also evaluated for known CAD risk factors, including diabetes mellitus (DM), arterial hypertension (AH), and body mass index (BMI). All patients underwent coronary angiography. The majority of patients were after primary percutaneous coronary intervention (PCI). Troponin I, lipid profiles, N-terminal pro-B-type natriuretic peptide (NT-pro-BNP), TH concentrations, and *DIO1, DIO2, DIO3* genetic polymorphisms were evaluated from a blood samples drawn before intervention procedures.

Follow-up data on mortality (time and cause of death) was used in the analysis as a primary outcome of interest. During a period of two-year follow-up, outcome data from 283 of the 290 participants was collected. The data was obtained from death certificates, post-mortem reports, and medical records. When data could not be obtained from these sources, the study team attempted to conduct telephone interviews with participant family members to obtain self-report mortality data or contacted the Causes of Death Register at the Institute of Hygiene of the Lithuanian Ministry of Health. Cardiac and all-cause mortality were ascertained. Documentation of death due to cardiac arrest or arrhythmias, death due to MI or progressive HF were regarded as cardiac-related mortality. The prospective study protocol was approved by The Regional Biomedical Research Ethics Committee and is described elsewhere^51^.

### Evaluation of TH and NT-pro-BNP

Blood samples were taken within 24 hours of patients’ admission to the ICU. The blood was centrifuged and the serum was frozen at –80° C. Serum samples were analysed in a single batch after completion of this study. Serum levels of T3, fT3, fT4, rT3 and TSH were analysed using an automated enzyme immunoassay analyser (Advia Centaur XP; Siemens Osakeyhtio). The normal range for total T3 was 0.89–2.44 nmol/L, fT3 3.50–6.5 pmol/L, fT4 11.50–22.70 pmol/L, rT3 24.50–269.30 pg/mL and TSH 0.55–4.78 mIU/L. The serum NT-pro-BNP levels were assessed using two-side chemiluminescent immunometric assay with Immulite 2000 immunoassay System; Siemens, Germany. All subjects included in the study were also evaluated for troponin I, lipid concentrations, serum glucose levels and underwent a common blood test.

### Genotyping

Six SNPs were evaluated for thyroid axes related genes including *DIO1* (rs11206244-C/T, rs12095080-A/G, rs2235544-A/C); *DIO2* (rs225014-T/C, rs225015-G/A) *DIO3* (rs945006-T/G). SNPs were selected if they were associated with serum TH levels in individual gene studies or based on data from Genome wide association studies^45,52,53^. We used minor allele frequency (MAF) of at least 10%. SNPs sequence in the studied genes - in *DIO1* gene locus rs11206244 (c.*29 C > T), rs12095080 (c.*1058 A > G), rs2235544 (c.682-34 C > A), *DIO2* gene locus rs225014 (p.Thr92Ala), rs225015 (c.*1453 C > T), *DIO3* gene locus rs945006 (c.*529 T > G). Information for genotyped SNPs is represented in Table 1. Genomic DNA was extracted from peripheral blood samples by the salting out procedure as described elsewhere^54^. The genotyping was completed using TaqMan SNP genotyping assays . (Applied Biosystems, Foster City, CA, USA): C_15952583_10 (rs2235544), C_31601225_10 (rs12095080), C_334342_20 (rs11206244), C_568127_10 (rs225015), C_15819951_10 (rs225014), C_7565113_10 (rs945006), and ABI 7900HT real-time PCR Thermocycler (Applied Biosystems, Foster City, CA, USA). Samples were measured in duplicates and nuclease-free water was used (AG00021000, 2114 BATCH 15595401, Sharlau, Spain) as no-template control.

**Table 1:** General information about genotyped loci for *DIO1, DIO2* and *DIO3* polymorphisms.

Gene/chromosome location	Polymorphism ID	Function	Variation	MAF^‡^	MAF
*DIO1*/1p32.3	rs11206244	3’UTR	c.*29 C > T	T = 0.313	T = 0.348
	rs12095080	3’UTR	c.*1058 A > G	G = 0.093	G = 0.081
	rs2235544	int3	c.682-34 C > A	A = 0.460	A = 0.481
*DIO2/*14q31.1	rs225014	missense, 3’UTR	p.Thr92Ala	C = 0.458	C = 0.279
	rs225015	3’UTR	c.*1453 C > T	A = 0.443	A = 0.260
*DIO3*/14q32.31	rs945006	3’UTR	c.*529 T > G	G = 0.189	G = 0.066

DIO – deiodinases, MAF^‡^ – reported minor allele frequencies in  single nucleotide polymorphisms databases from 1000 Genome Phase III combined population (http://www.ncbi.nlm.nih.gov/snp), MAF – minor allele frequencies in the present cohort, UTR – untranslated region; int – intron.

### Statistical analysis

Data is expressed as mean ± standard deviation (SD) for variables with Gaussian distribution and as median (25th–75th percentile) for variables without normal distribution. Normality of continuous data was assessed using the Kolmogorov-Smirnov test, analysis of the Q-Q plots and distribution in the histograms. Normal distribution was assessed and if necessary variables were natural-log transformed (ln). We specifically used a log transformation for NT-pro-BNP, TSH, and rT3 parameters.

Each SNP was tested for Hardy-Weinberg equilibrium (HWE) http://ihg.gsf.de/cgi-bin/hw/hwa1.pl ^55^, in case and contro l populations, using the Chi-square test or the Fisher’s exact test before inclusion in the association statistics (p > 0.01 threshold). Baseline clinical characteristics, TH levels, fT3 ranges (1^st^ quartile versus ≥2^nd^ quartile of fT3), NT-pro-BNP, and *DIO1, DIO2, DIO3* genotypes were compared across the cardiac-related death and survivors groups. Student’s t, Mann-Whitney’s U, Chi-square or Fisher’s exact tests were used to compare group scores as appropriate. Correlations between fT3, NT-pro-BNP were assessed using Pearson product-moment analysis (Pearson r). A p value <0.05 (two-tailed) was regarded as significant.

Univariate and multivariable Cox regression analyses were used to assess hazard ratio [HR] for all-cause and cardiac mortality. We made stringent attempts to control for the potentially confounding effect of (ln) NT-pro-BNP and other relevant sociodemographic and clinical factors such as age, Killip class, history of MI, history of hypertension, history of diabetes mellitus, history of chronic pulmonary disease and ST-elevation myocardial infarction. Kaplan-Meier survival curves for cardiac-related death and a log-rank (Mantel-Cox) test were employed for the analysis of survival curves. Statistical analyses was performed using the Statistical Package for the Social Science (SPSS23) for Windows.

## Results

### Baseline clinical characteristics, biomarkers levels and outcomes

Baseline demographics, clinical characteristics, CAD risk factors, concomitant disease, current treatment and concentration of biomarkers of 290 AMI patients are shown in Table 2. Two hundred and twenty four patients (77%) had AMI with ST-elevation, 66 (23%) with non ST-elevation, 236 (82%) had AH, 55 (19%) with DM and nine patients (3%) had chronic pulmonary disease. The majority of patients were Killip class II (74%), Killip class III (5%), and Killip class IV (3%). The mean of LVEF was 42.6 ± 9.8%. Eighty one percent of patients were taking beta-blockers, 92% – platelet antiaggregants, 89% – angiotensin-converting-enzyme inhibitors, and 10% – diuretics, and other medications.

**Table 2:** Sociodemographic, clinical characteristics and biomarkers of patients with acute myocardial infarction.

Characteristics	N = 290
Age (years), mean ± SD	62.0 ± 11.4
Body mass index, mean ± SD	29.9 ± 17.8
Systolic pressure (mmHg), mean ± SD	141.8 ± 25.9
Diastolic pressure (mmHg), mean ± SD	82.5 ± 13.5
Gender, n (%):	
Men	209(72.1)
Women	81(27.9)
Acute myocardial infarction type, n (%):	
With ST-segment elevation	224(77.2)
Non ST-segment elevation	66(22.8)
Myocardial infarction number, n (%):	
First	246(84.8)
Previous	44(15.2)
Killip class, n (%):	
I	53(18.3)
II	214(73.8)
III	15(5.2)
IV	8(2.7)
History of hypertension, n (%)	236(81.6)
History of diabetes mellitus, n (%)	55(19.0)
History of chronic pulmonary disease, n (%)	9(3.1)
Coronary Angioplasty and Stenting, n (%)	240(82.8)
Medications	
Nitrate, n (%)	238(82.1)
Beta-blockers, n (%)	235(81.0)
ACE inhibitors, n (%)	258(89.0)
Diuretics, n (%)	29(10.0)
Antiplatelet, n (%)	267(92.1)
Statins, n (%)	264(91.0)
Insulin therapy, n (%)	22(7.6)
N-terminal pro-B-Type natriuretic peptide (pg/mL), median (interquartile ranges)	1330.0(489.0–3461.0)
Thyroid-stimulating hormone (mIU/l), median (interquartile ranges)	1.00(0.6–1.5)
Free Thyroxine (pmol/l), mean ± SD	16.8 ± 2.7
Free Triiodothyronine (pmol/mL), mean ± SD	4.4 ± 0.7
Reverse Triiodothyronine (pg/mL), median (interquartile ranges)	646.9(489.5–1473.5)
Total Triiodthyronine (nmol/l), mean ± SD	1.6 ± 0.3

Values are presented as the mean ± SD, median (interquartile range), or percentage.

During the two-year follow-up period there were a total of 14 cardiac-related and 21 all-cause deaths. Patients in the cardiac-related death group were older, with more frequent cases of previous MI, a higher Killip class, a higher level of NT-pro-BNP, and more frequent cases of first quartile fT3 levels, as compared to survivors (Table 3). As well, there was a trend between first quartile of fT3 and higher cardiac-related mortality rates during first 30-days after a cardiac event (data not shown): patients with first quartile of fT3 consisted of older women with more severe HF (Killip class>I), followed by more cases of DM, higher NT-pro-BNP and troponin I levels, lower T3, reduced hemoglobin and hematocrit levels. Negative associations between fT3 and NT-pro-BNP (r = −0.30, p < 0.001) were established.

**Table 3:** Clinical characteristics of patients, who experienced cardiac death or survived due to MI.

Characteristics	Cardiac death	Survived	p-value
	n=14	n=269	
Age (years), mean ± SD	69.6 ± 8.4	61.3 ± 11.3	0.003
Body mass index, mean ± SD	30.7 ± 4.2	29.9 ± 18.3	0.603
Gender, n (%):			0.548
Men	9(64.3)	194(72.1)	
Women	5(35.7)	75(27.9)	
Myocardial infarction classification, n (%):			0.205
ST-elevation myocardial infarction	9(64.3)	211(78.4)	
Non-ST elevation myocardial infarction	5(35.7)	58(21.6)	
Myocardial infarction number, n (%):			
First	9(64.3)	230(85.5)	0.049
Previous	5(35.7)	39(14.5)	
Killip class, n (%):			0.004
I	1(7.1)	51(19.0)	
II	8(57.1)	202(75.1)	
III	4(28.6)	11(4.1)	
IV	1(7.1)	5(1.9)	
Hystory of Hypertension, n (%)	13(92.9)	217(80.7)	0.480
History of Diabetes mellitus, n (%)	5(35.7)	48(17.8)	0.149
History of Previous stroke, n (%)	2(14.3)	11(4.1)	0.130
History of Chronic renal disease, n (%)	1(7.1)	11(4.1)	0.463
History of Chronic pulmonary disease, n (%)	2(14.3)	6(2.2)	0.054
N-terminal pro-B-Type natriuretic peptide (pg/mL), median (interquartile ranges)	5104.0(1648.5–13863.0)	1238.0(475.0–3191.0)	<0.001
Thyroid-stimulating hormone (mIU/l), median (interquartile ranges)	1.1(0.5–2.4)	1.0(0.6–1.5)	0.773
Free Thyroxine (pmol/l), mean ± SD	17.4 ± 3.5	16.8 ± 2.6	0.579
Free Triiodothyronine (pmol/mL), mean ± SD	4.1 ± 0.8	4.4 ± 0.7	0.195
1^st^ quartile of Free Triiodothyronine versus ≥2^nd^ quartile of Free Triiodothyronine, n (%):			0.021
1^st^ quartile of Free triiodothyronine	7(58.3)	63(23.4)	
≥2^nd^ quartile of Free triiodothyronine	6(46.2)	206(76.6)	
Reverse Triiodothyronine (pg/mL), median (interquartile ranges)	941.8(329.5–1858.7)	635.6(491.5–1451.7)	0.849
Total Triiodthyronine (nmol/l), mean ± SD	1.6 ± 0.4	1.6 ± 0.3	0.855

Values are presented as the mean ± SD, median (interquartile range), or percentage. p-values are presented for Student’s t test, Mann-Whitney’s U test, the Chi-square test or Fisher’s exact test as appropriate.

### Association between deiodinases gene polymorphisms and cardiac mortality

Genotype distributions of all SNPs were found to be in HWE (p = 0.203 for rs11206244-C/T, p = 0.457 for rs12095080-A/G, p = 0.105 for rs2235544-A/C, p = 0.492 for rs225014-T/C, p = 0.677 for rs225015-G/A, p = 0.226 for rs945006-T/G). A relationship between gene polymorphisms and mortality was made in both cardiac mortality and survivor patient groups. Associations between *DIO1* (rs11206244-C/T, rs12095080-A/G and rs2235544-A/C), *DIO2* (rs225014-T/C, rs225015-G/A), and *DIO3* (rs945006-T/G) gene variants and cardiac mortality showed that in a case of assessed *DIO2, DIO3* polymorphisms, none of the SNPs were significantly associated with cardiac mortality in this AMI cohort.

However, the *DIO1* gene rs12095080 heterozygous AG genotype (OR = 3.97; 95% CI = 1.45–10.89; p = 0.005) showed a significant increased risk for cardiac-related mortality, while the major wild type homozygous AA genotype (OR = 0.26; 95% CI = 0.09–0.71; p = 0.006) was linked to increased survival. Allele analysis revealed that mutant G allele was significantly associated (OR = 3.31; 95% CI = 1.27–8.61; p = 0.036) with the risk of two year cardiac mortality (Table 4).

**Table 4:** Association between deiodinases genotype and two year cardiac-related mortality.

Gene	SNPs	Cardiac death	Survived	χ^2^	OR	95% CI	p-value
		n=14	n=269				
*DIO1*	*rs11206244*			0.437			0.803
	CC	7(50.0%)	111(41.4%)	0.403	1.390	0.501–3.857	0.526
	CT	6(42.9%)	130(48.5%)	0.170	0.805	0.287–2.261	0.680
	TT	1(7.1%)	27(10.1%)	0.128	0.698	0.095–5.136	1.000
	C allele	0.71	0.66	0.39	1.307	0.565–3.024	0.531
	T allele	0.29	0.34	0.39	0.756	0.331–1.771	0.531
	*rs12095080*			8.027			0.065
	AA	8(57.1%)	229(85.1%)	7.657	0.259	0.094–0.711	0.006
	AG	6(42.9%)	39(14.5%)	**8.003**	**3.967**	**1.446–10.885**	**0.005**
	GG	0	1(0.4%)	0.052	**—**	**—**	1.000
	A allele	0.79	0.92	6.66	0.302	0.116–0.788	0.036
	G allele	0.21	0.08	**6.66**	**3.306**	**1.269–8.610**	**0.036**
	*rs2235544*			3.162			0.226
	AA	1(7.1%)	66(24.7%)	2.263	0.246	0.003–1.844	0.200
	AC	8(57.1%)	146(54.7%)	0.033	1.100	0.392–3.086	0.857
	CC	5(35.7%)	55(20.6%)	1.810	2.046	0.712–5.879	0.179
	A allele	0.36	0.52	2.85	0.512	0.232–1.129	0.092
	C allele	0.64	0.48	2.85	1.955	0.886–4.313	0.092
*DIO2*	*rs225014*			2.899			0.248
	TT	10(71.4%)	134(49.8%)	2.488	2.413	0.775–7.514	0.170
	TC	4(28.6%)	115(42.8%)	1.098	0.551	0.177–1.716	0.408
	CC	0	20(7.4%)	1.120	**—**	**—**	0.609
	T allele	0.86	0.71	2.78	2.428	0.829–7.113	0.095
	C allele	0.14	0.29	2.78	0.412	0.141–1.206	0.095
	*rs225015*			2.209			0.312
	GG	10(71.4%)	143(53.2%)	1.788	2.124	0.682–6.613	0.181
	GA	4(28.6%)	108(40.1%)	0.746	0.611	0.196–1.900	0.577
	AA	0	18(6.7%)	1.000	**—**	**—**	0.610
	G allele	0.86	0.73	2.15	2.193	0.748–6.429	0.143
	A allele	0.14	0.27	2.15	0.456	0.156–1.337	0.143
*DIO*3	*rs945006*			0.009			1.000
	TT	12(85.7%)	233(86.6%)	0.009	0.931	0.217–3.997	0.923
	TG	2(14.3%)	36(13.4%)	0.009	1.075	0.250–4.615	1.000
	GG	0	0	**—**	**—**	**—**	**—**
	T allele	0.93	0.93	0.01	0.932	0.213–4.085	0.812
	G allele	0.07	0.07	0.01	1.073	0.245–4.700	0.812

DIO – deiodinases, SNP – single nucleotide polymorphism. Values are presented as number (percentage). p-values are presented for Hardy-Weinberg equilibrium (HWE) test and the Chi-square test or Fisher’s exact test as appropriate. Bold values: p-value <0.05 was regarded as significant.

*Source*: HWE: www.had2know.com/academics/hardy-weinberg-equilibrium-calculator.

### The prognostic importance of clinical variables, thyroid hormones, NT-pro-BNP and deiodinase genotypes on the mortality

Univariate regression analysis indicated that age, Killip class, NT-pro-BNP and history of chronic pulmonary disease were associated with all-cause mortality. The multiple Cox regression model showed no significant predictors of all-cause mortality (Table 5).

**Table 5:** Cox regression analysis for factors associated with all-cause and cardiac-related mortality.

Variable	Univariate		Multivariable	
	HR (95% CI)	p-value	HR (95% CI)	p- value
**All-cause mortality**				
Age	1.09(1.04–1.13)	<0.001	1.04(0.99–1.10)	0.097
Killip class	2.90(1.72–4.90)	<0.001	1.74(0.79–3.82)	0.167
Previous myocardial infarction	1.79(0.65–4.88)	0.258	0.92(0.28–2.99)	0.886
(ln) N-terminal pro-B-Type natriuretic peptide	1.92(1.35–2.72)	<0.001	1.45(0.93–2.26)	0.104
History of hypertension	2.25(0.52–9.67)	0.275	1.18(0.26–5.42)	0.837
History of diabetes mellitus	2.24(0.90–5.55)	0.082	1.48(0.50–4.41)	0.480
History of chronic pulmonary disease	5.89(1.73–50.01)	0.005	2.57(0.55–12.06)	0.233
ST-elevation myocardial infarction	2.12(0.88–5.10)	0.096	1.81(0.69–4.79)	0.230
Free Thyroxine	1.00(0.85–1.18)	0.987	0.88(0.75–1.02)	0.096
Free Triiodothyronine	0.55(0.26–1.18)	0.124	0.69(0.34–1.41)	0.691
1^st^ quartile of Free Triiodothyronine versus ≥2^nd^ quartile of Free Triiodothyronine	2.07(0.85–5.07)	0.111	1.57(0.58–4.26)	0.371
*DIO1*rs12095080	0.45(0.17–1.16)	0.096	0.52(0.19–1.46)	0.214
rs12095080 AG	2.23(0.87–5.75)	0.096	1.94(0.69–5.47)	0.211
rs12095080 AA	0.45(0.17–1.16)	0.096	0.52(0.18–1.46)	0.212
1^st^ quartile of Free Triiodothyronine versus ≥2^nd^ quartile of Free Triiodothyronine & rs12095080	1.66(1.09–2.51)	0.018	1.41(0.90–2.20)	0.131
**Cardiac-related mortality**				
Age	1.07(1.02–1.13)	0.008	1.01(0.95–1.08)	0.668
Killip class	2.94(1.53–5.67)	0.001	1.79(0.71–4.48)	0.217
Previous myocardial infarction	3.06(1.02–9.14)	0.045	1.43(0.40–5.17)	0.582
(ln) N-terminal pro-B-Type natriuretic peptide	2.37(1.50–3.74)	<0.001	2.11(1.18–3.78)	0.012
History of hypertension	3.09(0.40–23.61)	0.277	1.47(0.18–12.20)	0.722
History of diabetes mellitus	2.50(0.84–7.46)	0.101	2.11(0.59–7.58)	0.252
History of chronic pulmonary disease	6.39(1.43–28.58)	0.015	3.61(0.61–21.30)	0.157
ST-elevation myocardial infarction	1.97(0.66–5.87)	0.226	2.27(0.65–7.95)	0.199
Free Thyroxine	1.08(0.89–1.29)	0.446	1.01(0.95–1.08)	0.652
Free Triiodothyronine	0.43(0.16–1.14)	0.089	0.82(0.28–2.36)	0.547
1^st^ quartile of Free Triiodothyronine versus ≥2^nd^ quartile of Free Triiodothyronine	3.57(1.20–10.62)	0.022	2.30(0.68–7.73)	0.180
*DIO1*rs12095080	0.25(0.09–0.71)	0.009	0.32(0.10–1.09)	0.069
rs12095080 AG	4.09(1.42–11.78)	0.009	3.14(0.92–10.72)	0.069
rs12095080 AA	0.25(0.09–0.71)	0.009	0.32(0.09–1.09)	0.069
1^st^ quartile of Free Triiodothyronine versus ≥2^nd^ quartile of Free Triiodothyronine & rs12095080	2.40(1.48–3.92)	<0.001	1.74(1.04–2.91)	0.034

aMultiple Cox regression analyses adjusted for age, Killip class, previous myocardial infarction, (ln) N-terminal pro-B-Type natriuretic peptide, history of hypertension, history of diabetes mellitus, history of chronic pulmonary disease, ST-elevation myocardial infarction.

Univariate regression analysis indicated that age, Killip class, previous MI, NT-pro-BNP, history of chronic pulmonary disease as well as first quartile versus ≥ second quartile of fT3 and *DIO1* gene rs12095080 were all significantly associated with cardiac-related mortality. Furthermore, after adjustment for clinical and demographic variables, the multiple Cox regression model showed that NT-pro-BNP (HR = 2.11; 95% CI = 1.18–3.78; p = 0.012) and first quartile of fT3, and *DIO1* gene rs12095080 are significant risk factors for cardiac-related mortality (HR = 1.74; 95% CI = 1.04–2.91; p = 0.034) after AMI (Table 5).

**Figure 1:**
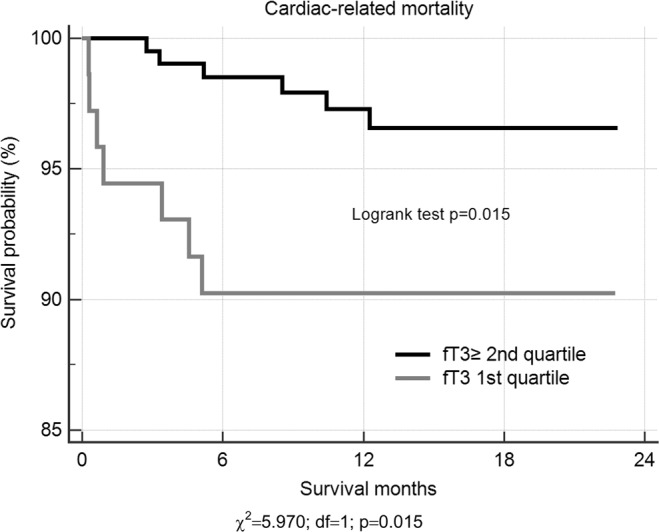
Two year Kaplan-Meier survival curves for cardiac-related death in patients with AMI stratified on fT3 quartiles. A log-rank test was used to compare survival curves.

**Figure 2:**
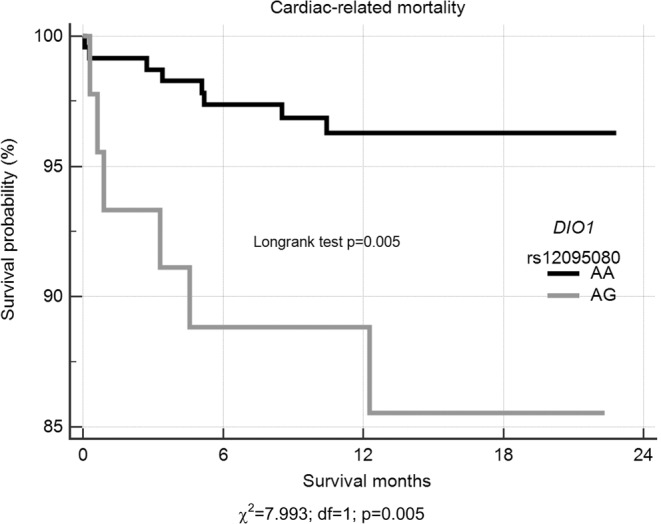
Two year Kaplan-Meier survival curves for cardiac-related death in patients with AMI according to *D1O1*rs12095080 genotypes. A log-rank test was used to compare survival curves.

Kaplan-Meier two-year survival curves stratified on fT3 levels, according quartiles, provided significant prognostic information. The highest risk for cardiac mortality was among AMI patients within the first quartile of fT3, compared to patients with all other quartiles (HR = 3.57; 95% CI = 1.20–10.62; p = 0.022) (Fig. 1). Moreover, Kaplan-Meier analyses showed decreased length of survival in a group of *DIO1* gene rs12095080 AG genotype carriers (HR = 4.09; 95% CI = 1.42–11.78; p = 0.009) (Fig. 2). Patients carrying rs12095080 heterozygous genotype were found to experience death 2.5 months earlier (19.7 ± 1.0 months vs. 22.2 ± 0.23 months; log-rank χ^2^ = 7.99, p = 0.005), as compared to AA genotype carriers (Fig. 2).

## Discussion

In this research study we aimed to explore possible associations between serum levels of TH, genetic polymorphisms of *DIO*, and NT-pro-BNP with long-term outcomes in AMI patients.

It was found that lower fT3 levels, *DIO1* gene rs12095080, as well as higher NT-pro-BNP on admission are all associated with cardiac-related mortality after AMI. The hypothesis proposing that

variations in TH concentrations within the statistically normal range may influence disease outcomes is not entirely new^26,56,57^. Nevertheless, a low T3 syndrome does not only reflect AMI status, but it has also been documented in a number of other disorders^58–61^.

Independent of time-course, type and severity, a low T3 state may serve as an adaptive mechanism which reduces metabolic demands by reducing the catabolic processes of the disease^8^. A low T3 syndrome was a frequent finding in patients with cardiac pathology and without a history of thyroid dysfunction, particularly among patients with HF, AMI, and those following cardiac surgery^15–17,62–65^. However, the exact point of occurrence of THs alterations, after an ACS, is not clearly understood^2,66–68^. Timing of TH alterations is still debated topic in the scientific literature. However, most of the studies agree that the first five days of ACS are the most crucial for changes in T3 and rT3. Iltumur *et al*.^69^ observed that patients with complicated MI (caused by ischemia) have a lower total and fT3. Besides, patients with prolonged cardiac arrest showed lower total T3 and fT3 levels than those with shorter one. Furthermore, during the AMI stage, drugs like nonsteroidal anti-inflammatory agents, aspirin, heparin and furosemide (>80 mg/day) might have an effect of displacing T4 and T3 from TH binding sites on TH binding proteins, which modify hormone delivery to the location of its use^70,71^.

Our study findings correspond to the findings of Zhang *et al.*^17^ exemplifying that patients with AMI and with first quartile of fT3 levels, are more likely to be older women, with severe HF (Killip class>I), followed by DM. Our study AMI patients also had a higher level of troponin I, lower T3, as well as lower hemoglobin and hematocrit levels. The low T3 pattern pathophysiological role is not well understood, although high mortality among patients with low T3 levels is found in numerous studies^1,12,17,37,63^. Conversely, other studies have not discovered an independent prognostic role for low T3 levels in cardiovascular patients^72–75^. Our study revealed a decreased length of survival in AMI patients with first quartile of fT3, confirming previous findings. Additionally, we estimate that fT3 levels within the normal concentration ranges was probably due to omitted analysis of TH during the later post-AMI period when greater fT3 downregulations could be observed^2,16,18,66–68,76^.

The present study lends support to the theory advanced by other research teams that fT3 represents the biologically active form of TH, so an isolated reduction in its level could constitute a model of abnormal TH metabolism acting as a risk factor for CAD^3,27–29^. Further, subclinical hypothyroidism, characterized by normal serum concentrations of fT4 and elevated TSH showed as a predictor of atherosclerosis and MI risk in elderly women^3,27,77,78^. It is suggested that even within the clinically normal range variations of TH indicate abnormal TH metabolism associated with coronary disease risk and outcomes^24,27–30,79^. However, Ertas *et al*.^28^ showed that within the normal range fT3 levels were inversely associated with CAD severity. It was also found that lower fT3 concentrations independently predicted the severity of CAD^29^. Mayer *et al*., showed that even minor changes of fT4 may relate with severity of HF^30,31^. fT4 serum concentration levels association with coronary disease severity was also examined in Jung *et al*.’s study^26^. When compared with survivors patients that died within seven days after AMI had a higher fT4 level, thus it is possible to make an assumption that higher levels of fT4 might be associated with increased survival rate^2,25^. Our present and previous studies and those of others, indicate association between fT3 or low-T3 syndrome with elevated NT-pro-BNP levels. This is a traditional predictor of poor prognoses in patients with AMI, indicating that a lower fT3 level would be a predictor of a poor prognosis in CAD and AMI patients^17,23,80,81^. The current study also presented a negative association between fT3, NT-pro-BNP levels and CAD outcomes which was confirmed by others authors^80–83^.

There are several well-known TH-pathway genes such as *DIO*, TSH receptor (THR), and TH transporters (*SLCO, MCT*), which have been associated with TH levels^84^. Variants in both *DIO1* and *DIO2* genes were recently reported to alter TH levels in healthy individuals^34,45,85,86^. TH metabolism roles are determined by three iodothyronine deiodinases DIO1, DIO2 and DIO3 encoded by a separate gene^37,38,40,87^. The *DIO1*, which is responsible for converting T4 into T3, and contributes to the local hypothyroid state in the failing heart^4,12,37^. It was shown, experimentally, that alterations in *DIO1* and *DIO2* promote cardiac activity of *DIO3*, converting T4 and T3 to inactive reverse T3 and diiodothyronine (T2) in rats following MI^88^. Altered thyroid homeostasis in patients with cardiovascular disorders could modify cardiac gene expression and contribute to impaired cardiac function^89,90^. A candidate gene study revealed rs2235544 in *DIO1* gene was associated with higher fT3 and lower fT4 and rT3 levels in both patients receiving TH replacement therapy and in a large population of healthy individuals. Rare C allele was associated with improved *DIO1* function^44,52^. Several studies identified rs11206244 in *DIO1*, which was also associated with fT4, rT3 and fT3 concentrations^34,91^. Numerous studies disclosed an association between *DIO1, DIO2, DIO3* polymorphisms and fT3 and other TH levels^33,34,42,92^. Our data of the same cohort also endorsed that *DIO1, DIO2* gene polymorphisms are mainly associated with T3, fT4, fT3/fT4, (ln)rT3 levels, while organic anion transporter polypeptide *1C1* rs1515777-AG minor allele homozygous genotype was associated with a decrease in circulating fT3, fT3/fT4 in CAD patients after AMI^46^.

Genetic variations in deiodinases may affect multiple clinical endpoints^36,37,42,93^. It was shown that the development of CAD is the result of complex interactions between numerous environmental factors and genetic variants at many loci^94,95^. In our previous study we found that *DIO1* rs12095080 was associated with AH, while *DIO2* rs225015 was associated with DM, and SNP rs974453-genotypes was associated with STEMI within the *OATP1C1* gene^46^.

Lee *et al*. found that cardiovascular mortality was higher in subjects with the rs4977574 GG genotype than in those with other genotypes^96^. The association between four SNPs on chromosome 9p21, CAD, and MI has been replicated several times in multiple populations^97–100^ In patients with MI with ST-segment elevation Szpakowicz *et al*. revealed association between the rs12526453 of the phosphatase and actin regulator 1 (PHACTR1) gene and 5-year mortality^101^. However, in another study, the DIO2 Thr92Ala polymorphism was not related with thyroid parameters, cognitive functioning and health-related quality of life^102^. In the present study we found a relationship between SNPs in *DIO1* gene rs12095080 heterozygous genotype (AG) and cardiac-related mortality. It should be noted that no patients in the cardiac-related death group carried the homozygous mutant GG genotype of this SNPs. Patients carrying rs12095080 heterozygous genotype experienced 2.5 months shorter median survival as compared to AA genotype carriers. Our preliminary analysis shows that G allele could be a favourable variable to investigate for AMI patient’s prognosis. To our knowledge, there are no reports showing the importance of fT3 ranges and genetic variability of *DIO1* in the long-term outcomes of the patients with AMI. There is evidence that the G variant in rs12095080, identified in the 3’ UTR of human *DIO1* mRNA, is associated with higher T3/rT3 ratio in serum. This may suggest that some variants in this SNPs may result in increased *DIO1* activity^103^. Palmer *et al*.^104^ showed that angiotensin-converting enzyme genotype powerfully predicted mortality in patients after AMI. They also showed that the ACE genotype DD was positively associated with the B type natriuretic peptide and was an independent predictor of death and the effects the response to treatment^105^.

To our knowledge this study is the first one to examine how concentrations of TH and genetic markers in patients after AMI might contribute to long term outcomes. However, our findings are still exploratory and it would be premature to use them as a basis for risk stratification in patients with CAD. For example, future studies are needed to explore fT3 and gene polymorphism mutual interaction on the underlying cardiovascular mortality mechanisms. Understanding the genetic factors contribution to TH expression that predict cardiac-related mortality may open new markers and treatment targets for management of cardiovascular disease. For example, as suggested by Pingitore *et al.*^18^ by knowing the exact mechanism we might not only measure fT3 concentration in patients after an AMI and patients with multiple CAD risk factors but also treat those with low fT3 and see whether their clinical outcomes improve.

The main limitation of this study is that clinical research was performed in a single centre with a limited number of subjects. These results require validation in studies that replicate the model and include a higher number of cases and controls. Additionally, the majority of studied AMI patients had mild to moderate HF and we did not include other risk factors in our study, such as left ventricular ejection fraction or smoking. Thus, the results presented may be limited in their generalizability and may not apply to patients with more advanced HF.

Finally, baseline levels of TH were not evaluated in this study, as TH was measured only on admission to the ICU and was not investigated during the later post-AMI period when the hormone concentration decline is lasting^2,66–68^. The strengths of this study include its novelty – the assessment of an impact of the fT3 ranges and TH gene polymorphisms on long-term mortality while controlling for disease severity and other CAD risk factors in patients with AMI.

## Conclusions

Lower fT3 level and *DIO1* gene rs12095080 as well as higher NT-pro-BNP on admission are associated with cardiac-related mortality after AMI. In a case of *DIO1* gene rs12095080, heterozygous AG genotype was significantly associated with a higher risk for cardiac mortality. Conversely, major wild type homozygous AA genotype was linked to better survival within the two year follow-up period.

### Ethics approval and consent to participate

The study and its consent procedures were approved by the Kaunas Regional Biomedical Research Ethics at Lithuanian University of Health Sciences, Kaunas, Lithuania and conform to the principles outlined in the Declaration of Helsinki. Written informed consent was obtained from each study patient.

## Supplementary information

Supplementary inforamtion.

## Data Availability

The datasets analysed during the current study are available from the corresponding author upon request
