# Supplementary material for: Importance of Thyroid Hormone level and Genetic Variations in Deiodinases for Patients after Acute Myocardial Infarction: A Longitudinal Observational Study
Source: Sci Rep. 2020 Jun 8;10:9169. doi: 10.1038/s41598-020-66006-9 (PMC7280282; doi:10.1038/s41598-020-66006-9)
Supplement: Supplementary file 1 — Supplementary inforamtion. [file 41598_2020_66006_MOESM1_ESM.pdf]

Gene-Environment Interactions Connecting Low Triiodothyronine Syndrome and Outcomes of Cardiovascular Disease (GET-VASC)

Julius BURKAUSKAS[1], Narseta MICKUVIENĖ[1], Julija BROŽAITIENĖ[1], Margarita STANIŪTĖ[1], Aurelija PODLIPSKYTĖ[1], Daiva RASTENYTĖ[2], Olivija GUSTIENĖ[2], Henrikas KAZLAUSKAS[1], Robertas BUNEVIČIUS[1]

[1] Lithuanian University of Health Sciences Institute of Behavioral medicine, Palanga, Lithuania;

[2] Hospital of Lithuanian University of Health Sciences Kaunas Clinics, Kaunas, Lithuania

SUMMARY

Background. The mortality rate in Lithuania is among highest in Europe and cardiovascular mortality covers about 56% of the general mortality. Survivors after myocardial infarction (MI) often demonstrate symptoms, modifiable factors and biomarkers that affect both mortality and quality of life after acute cardiovascular events. It is an important task to understand and improve outcomes in cardiovascular disease. Decreased triiodothyronine (T_3) concentration is observed in patients after an acute MI which has been linked to adverse prognosis and to co-morbidities such as depression. Genetic alterations in various components of thyroid hormone signaling and environmental factors such as dietary selenium or iodine are associated with morbidity, mortality and well-being of patients. However, these interactions in patients after acute cardiovascular events have not been studied. Better understanding of thyroid hormone signaling in brain-thyroid-heart interaction may open new markers and new targets for managing MI as well as depression.

The aim of the research. This research project aims to establish whether polymorphism in thyroid axis related genes interacting with environmental factors affect thyroid hormone concentrations, survival and well-being of patients after acute MI.

Methods/Design. We plan to recruit patients after acute MI in cross-sectional and in follow-up design to evaluate if socio-demographic, clinical, psychological, endocrine, environmental and genetic factors affect survival and health related quality of life after major cardiovascular events.

Discussion. We expect that the project will give important new knowledge in understanding mechanisms by which gene and environmental factors are associated with cardiovascular events, and by which they affect outcomes and well-being of patients. It will also provide a pharmacogenetic foundation for new clinical trials evaluating endocrine, cardiovascular and dietary interventions in patients after acute MI.

INTRODUCTION

The mortality rate in Lithuania is among highest in Europe [1] and in 2010 it was 1281/100000. Cardiovascular mortality covers about 56% of general mortality. Coronary artery disease and acute myocardial infarction (MI) cause 62% of cardiovascular mortality. Survivors after acute MI often demonstrate symptoms of depression [2, 3], fatigue [4, 5], poor cognitive functioning [6] and worsening health related quality of life [7, 8]. Finding modifiable factors and biomarkers that affect both mortality and well-being of patients after acute cardiovascular events is an important task to further understand and improve outcomes in cardiovascular disease (CVD).

A decrease in triiodothyronine (T_3) concentrations, so called low T_3 syndrome or non-thyroid illness syndrome, is observed in about 1/3 of patients after acute cardiovascular events and has been linked to the severity of the disease [9], to its adverse prognosis, including development of complications such as heart failure, and to co-morbidities such as depression and fatigue [10]. However, the exact origin of this syndrome is not clear. Both poor secretion of thyrotropin-releasing hormone (TRH) in the hypothalamus and suppression of tissue T_3 conversion from less active thyroxine (T_4) by enzymes called deiodinases were demonstrated to be involved in the development of low T_3 syndrome [11]. The need for replacement therapy in low T_3 syndrome is controversial. However, one study in heart failure patients has demonstrated improvement in cardiovascular function and endocrine profile of patients treated with T_3 [12, 13].

Genetic alterations in various components of thyroid hormone signaling such as deiodinase (DIO1, DIO2, DIO3), thyroid hormone transporters (OATP1c1) and thyroid hormone receptors (TR-alpha, TR-beta) were demonstrated to be associated with thyroid hormone concentrations in serum, as well as with symptoms of depression and fatigue in patients with hypothyroidism and in patients with depression [14]. Moreover, pharmacogenetic studies have demonstrated that adjunct treatment with T_3 improves well-being of hypothyroid patients and mood in depressed patients, but only in those with specific genetic polymorphism [15]. However, there is no evidence, in patients after acute cardiovascular events, to show if manifestation or compensation of low T_3 syndrome is associated with any genetic markers.

On the other hand, environmental factors, such as dietary iodine and selenium, may affect thyroid hormone concentrations in serum and tissues. Iodine is an important component for the synthesis of thyroid hormones. Selenium is a key-element for the deiodinases, selenoenzymes responsible for the tissue thyroid hormone metabolism. It is well known that iodine sufficiency is important for brain development. It was demonstrated that low serum selenium concentrations in severe illness lead to impaired T_4 and T_3 metabolism. Substitution of selenium in acute illness improves morbidity, and in thyroid disease reduces autoimmunity and improves quality of life [16]. However,

there are no data on effects of iodine or selenium deficiency or substitution on outcomes and well-being of patients after acute cardiovascular events.

We have demonstrated that low T_3 concentrations after acute cardiovascular events may be a mechanism connecting depression, fatigue and quality of life with CVD. Depression is common and most widely studied psychiatric condition in patients with CVD. Increased rates of depression are reported after acute MI and in congestive heart failure (HF). Depression is a risk factor for development of CVD, worsening the prognosis, increasing disability, reducing quality of life, and increasing mortality in patients following MI and stroke. In CVD patients depression doubles mortality. In post-MI patients it more than doubles mortality. Several studies have reported a dose-response relationship between the severity of depression and prognosis of CVD [17-21].

In sum, understanding the genetic and environmental factors that predict development of low T_3 syndrome may open new markers and treatment targets for management of CVD and related co-morbidities such as depression. It is also important to investigate the mechanisms by which low T_3 syndrome affects survival and well-being of CVD patients.

Aim and objectives

The aim of this research project is to establish whether polymorphism in thyroid axis related genes interacting with environmental factors affect thyroid hormone concentrations, survival and well-being of patients after acute MI. The objectives of the study will be to:

1. To determine whether genetic polymorphism of thyroid axis related proteins or environmental factors such as iodine and selenium insufficiency or their interaction are related with low T_3 syndrome in patients after acute MI.

2. To determine whether clinical outcomes of acute MI are related with the presence of low T_3 syndrome or with genetic polymorphism of thyroid axis related proteins or with environmental factors such as iodine and selenium insufficiency.

3. To determine whether depression and cognitive functioning of patients after MI are related with low T_3 syndrome or with genetic polymorphism of thyroid axis related proteins or with environmental factors such as iodine and selenium insufficiency.

4. To determine whether gene-environmental interactions concerning manifestation of low T_3 syndrome, survival and well-being of patients after acute MI can be extrapolated to stroke patients.

METHODS

GET-VASC is a multicenter, cross-sectional prospective study. The sponsor of this biomedical research is the Lithuanian University of Health Sciences (LUHS). The study is performed at the LUHS Kaunas Hospital Department of Cardiology and LUHS Institute of Behavioral Medicine[1].

We performed power and sample size calculations for the primary aim of the study hypothesizing that prevalence of DIO1 polymorphism and its impact on T_3 serum concentrations will be not lower than in the Rotterdam elderly population [22]. With a significance level of 0.05 and one-tailed test we will need approximately 220 total subjects to achieve 80% power. Use of selenium concentrations as covariates may increase or decrease statistical power. Therefore, inclusion of 400 patients will leave us sufficient insurance to be able to detect effect sizes smaller than we have predicted. Moreover, we expect that the genetic and environmental impact on T_3 concentrations after stress caused by the acute cardiovascular event will be more evident than in normal conditions

We anticipate, based on the experience of our cardiologists that of the first 500 consecutive patients who will meet the inclusion criteria of the study, about 10% will not be interested in participating. Among 450 patients who, as we predict, will sign informed consent about 11% (about 50 patients) will be excluded from the analyses due to abnormal serum thyrotropin concentration (undiagnosed clinical or subclinical hyperthyroidism or hypothyroidism) or due to presence of TPO antibodies. Majority of excluded patients will be women because autoimmune thyroid disease and thyroid dysfunction are more prevalent among women. This study will include a moderate group of patients, making it necessary to study a relatively homogeneous sample for statistical analysis reasons.

Inclusion criteria:

- Acute MI
- Able to understand and provide written informed consent
- Patients 18-80 years of age
- Patients of any ethnic background but fluent in Lithuanian.

Exclusion criteria:

- Hypothyroidism or hyperthyroidism, according to increased or suppressed thyrotropin concentrations (normal range from 0.5 to 3.7 μg/mL),

[1] The name of the Institute of Behavioral medicine of Lithuanian University of Health Sciences has been changed to Laboratory of Behavioral Medicine, Neuroscience Institute, Lithuanian University of Health Sciences from the1st of January, 2018

- Autoimmune thyroid disease, according to increased concentration of TPO antibodies (>35 U/mL).
- Treatment with thyroid hormones, anti-thyroid medication or amiodarone within 3 weeks.
- Use of corticosteroids
- Severe concomitant diseases, such as renal failure, liver cirrhosis, cancer, active infection, decompensated diabetes
- Severe cognitive dysfunction (dementia or delirium)
- Pregnant.

During screening, a patient who meets the inclusion criteria is invited to participate in the study and sign an informed consent form. The study is conducted in four stages: the screening phase, the clinical evaluation phase, evaluation of rehabilitation program and the long-term follow-up phase. The content of each step will consist of observations that are relevant to that step of treatment. The stages of evaluation include: clinical evaluation and socio-demographic characteristics, functional status testing, anthropometric measurements, questionnaire completion, diagnostic interviews, and psychological tests. Blood is drawn for biochemical, micronutrient, endocrine and genetic testing.

Experimental design and methods

The protocol of the study will be approved by the by the Kaunas Regional Biomedical Research Ethics Committee. Patients during the hospitalization phase will be tested at the study centers (hospitalization day 1, hospitalization day 4 and rehabilitation day 14-21). If patients are discharged from the hospital (day 4 or day 7) they will be invited to visit the Cardiology outpatient department at the LUHS Kaunas Hospital. Rehabilitation of the patients will be provided at the Department of Rehabilitation of Institute of Behavioral Medicine, LUHS[1].

Visit 1. Day 1, Acute phase. After arrival patient will receive standard evaluations that include:

- Routine clinical evaluation including Killip classification, blood pressure (BP) and electrocardiogram (ECG).
- Approximate time of MI.
- Routine biochemical data: peripheral blood, K^+, Na^+, creatinine, glucose, activated partial thromboplastin time (APTT) and troponine I, collected from the medical documentation.
- Routine echocardiographic data of left/right ventricle performance, ejection fraction, status of heart valves collected from the medical documentation.
- Additional blood samples will be drawn to assess thyroid axis hormones concentrations, inflammation factors, selenium and NTpro-BNP.

- Additional blood samples drawn for genetic evaluations for thyroid axis related genes and for depression related genes.
- Cognitive tests for delirium evaluated using Confusion Assessment Method instrument.

Visit 2. Day 4, Sub-acute phase. At the Department of Cardiology alongside with standard routine clinical and ECG re-evaluation additional research procedures will be performed:

- EuroQOL five dimensions questionnaire (EQ-5D).
- Pittsburgh Sleep Quality Index (PSQI).
- Hospital Anxiety and Depression Scale (HADS).

Visit 3. Day 12-21. Rehabilitation phase. At the Department of Rehabilitation alongside with standard routine clinical and ECG re-evaluation, and exercise test additional research procedures will be performed:

- Evaluations of functional status (veloergometry, ECG and BP monitoring)
- MINI Neuropsychiatric Interview for diagnoses of mood and/or anxiety disorders, Beck Depression Inventory (BDI).
- Comprehensive battery for cognitive evaluations.
- Quality of life assessment (Minnesota Living with Heart Failure Questionnaire, MLHFQ; the 36-item Short Form Medical Outcome Questionnaire, SF-36).
- EuroQOL five dimensions questionnaire (EQ-5D).
- Pittsburgh Sleep Quality Index (PSQI).
- Ten Item Personality Inventory (TIPI).
- Blood samples for thyroid axis hormones, hsCRP and NTpro-BNP will be drawn.

Visit 4. Follow-up phase (2 years follow up). Telephone calls every half a year for:

- Minnesota Living with Heart Failure Questionnaire (MLHFQ).
- EuroQOL five dimensions questionnaire (EQ-5D).
- Information on mortality and re-infarction.

Clinical evaluation

Acute MI diagnoses is established by typical ischemic chest pain, by presence of ST - elevation on the ECG (ST-elevation ≥ 2 mm in ≥ 2 consecutive precordial leads, or ≥ 1 mm in ≥ 2 consecutive standard limb leads), and with non ST-elevation and by increased troponin concentration.

The severity of the MI will be assessed by the routine physical examination derived parameters using the Killip classification, dividing patients into classes I to IV (I, no heart failure; II, some evidence of such a complication; III, pulmonary edema; and IV, cardiogenic shock).

Data on routine biochemical evaluations will be collected from the medical documentation. For additional tests (hormone concentrations) blood will be drawn at the acute phase of MI, centrifuged and serum is frozen and stored at the -80°C. All serums will be assessed during one evaluation to avoid inter-assay variability. Concentrations of thyroid axis hormones (TSH, FT_3, FT_4, rT_3), inflammation factors (hsCRP) and NTpro-BNP will be assessed.
For genetic evaluations blood will be drawn at the acute phase of MI. Blood samples will be frozen and stored at the -80°C. Common polymorphisms for thyroid axes related genes, deiodinase-1 (DIO1), deiodinase-2 (DIO2), deiodinase-3 (DIO3), and organic anion transporter polypeptide 1c1 (OATP1c1) will be assessed.

Diagnostic psychiatric interview

Study patients will be interviewed for the psychiatric diagnosis using the MINI International Neuropsychiatric Interview (MINI; see Appendix) [23]. This instrument is a structured diagnostic interview for diagnosis of mental disorders using the criteria of the Diagnostic and Statistical Manual of Mental Disorders (DSM-IV-TR) and the ICD-10 Classification of Mental and Behavioral Disorders. MINI has a modular structure including a screening module and modules for specific mental disorders. In this study a screening module and modules to assess current and past diagnoses of mood disorders, such as major depression (Module A), dysthymia (Module B) and module for current suicidality (Module C) will be used. We will use diagnosis of current depressive disorder, not past depressive disorder, as the main diagnostic variable. The modular structure of the MINI allows the investigator to skip specific modules if the subject does not meet the screening criteria [24].

Cognitive functioning

Cognitive functioning will be assessed by the Mini Mental State Examination (MMSE), a tool that can be used to systematically and thoroughly assess cognitive status. It is an 11-question measure that tests five areas of cognitive function: orientation, registration, attention and calculation, recall, and language. The maximum score is 30. A score of 23 or lower is indicative of cognitive impairment including depression or delirium [25, 26].

Questionnaires

After a diagnostic interview, patients will be asked to complete study questionnaires.
The Hospital Anxiety and Depression Scale (HADS) [27]. The HADS comprises 14 items scored from 0 to 3 to which patients respond based on their experience over the past week. Seven items

comprise a subscale for depression with a score range from 0 to 21; seven other items comprise a subscale for anxiety. The HADS is a self-rating scale for non-psychiatric clinical settings and is frequently used in cardiac clinics [28, 29]. A cut-off value of > 10 defines patients with severe symptoms of depression or anxiety [30].

The Beck Depression Inventory (BDI), will be used to screen for depressive disorder and to provide a measure of severity of depressive symptoms [31]. The BDI consists of 21 items, with a score range from 0 to 63, in which scores less than 10 indicate normal mood variation and scores of 10 or greater reflect increasing degrees of depression [28].

The Ten Item Personality Inventory (TIPI) is a 10-item measure of the ‘big-five’ personality dimensions, including openness to experience, conscientiousness, extraversion, agreeableness, and neuroticism [32]. Each scale provides two items rated on a 7 point scale from ‘strongly disagree’ to ‘strongly agree’. The TIPI is offered for situations where very short measures are needed and personality is not the primary topic of interest. Personality traits such as neuroticism are potential confounders in depression and cardiac patients.

The Minnesota Living with Heart Failure Questionnaire (MLHFQ) [33] consists of 21 questions covering physical, socioeconomic and psychological dimensions of life, related to limitations frequently associated with the profile of cardiac insufficiency. This health-relation quality of life (HRQoL) measure was chosen over generic assessment scales as it specifically addresses the daily living challenges of individuals with heart failure. 26 Items are rated on a six-point Likert scale from 0 (‘no impact of heart failure on HRQoL’) to 5 (‘significant negative impact of heart failure on HRQoL’).

The 36-item Short Form Medical Outcome Questionnaire (SF-36) [34] consists of 8 multi-item subscales that assess HRQoL on 8 domains: physical functioning, social functioning, role limitations due to physical problems, role limitations due to emotional problems, mental health, energy/vitality, pain, and general health perception. Each of the 8 SF-36 domains is scored on scales from 0 to 100, with higher scores indicating better health-related quality of life. Internal reliability (α coefficients) of 8 subscales has been found to range between .56 and .85.

EuroQOL five dimensions questionnaire (EQ-5D) [35] comprises two parts: the EQ-5D self-classifier, a self-reported description of health problems according to a five-dimensional classification (i.e., mobility, self-care, usual activities, pain/discomfort and anxiety/depression) and the EQ visual analogue scale (VAS), a self-rated health status using VAS, to record the perceptions of a participant’s current overall health; the scale ranges from 0 (the worst health state) to 100 (the best health state).

Multidimensional Fatigue Inventory (MFI-20) [36] measures subjective fatigue level for starting any activity using a 20 item scale. Patients rate how well each statement about fatigue applies to them on a 5-point Likert scale, based on their experience during the past few days ranging from 1 (no fatigue) to 5 (high level of fatigue). Possible scores on MFI-20 on each subscale range from 4 to 20, with a higher score indicating higher levels of fatigue.

The Patient Health Questionnaire (PHQ) [37] assesses 8 diagnoses, divided into threshold disorders (disorders that correspond to specific DSM-IV diagnoses: major depressive disorder, panic disorder, other anxiety disorder, and bulimia nervosa), and subthreshold disorders (disorders whose criteria encompass fewer symptoms than are required for any specific DSM-IV diagnoses: other depressive disorder, probable alcohol abuse/dependence, somatoform, and binge eating disorder).

The Pittsburgh Sleep Quality Index (PSQI) [38] is a self-rated questionnaire which assesses sleep quality and disturbances over a 1-month time interval. Nineteen individual items generate seven "component" scores: subjective sleep quality, sleep latency, sleep duration, habitual sleep efficiency, sleep disturbances, use of sleeping medication, and daytime dysfunction. The sum of scores for these seven components yields one global score.

Data on mortality will be collected via telephone calls and via the Causes of Death Register at the Institute of Hygiene of the Lithuanian Ministry of Health.

General Statistical Analysis Considerations

Data will be stored and analyzed at the Institute of Behavioral Medicine, LUHS[1]. This information will be protected by password known only to investigators working on this protocol.

Preliminary Exploration: We will explore the data using descriptive statistics prior to any hypothesis testing. For categorical variables we will examine frequency distributions and where appropriate contingency tables and histograms. For continuous variables, we will examine frequency distributions and where appropriate plots. When appropriate, we will consider transformation. If necessary due to distributional considerations, we will consider a change of analysis method to a less parametric one.

General Modeling: The design is a two-by-two factorial design, and depending on the response variable, the analysis method will be either ANOVA or logistic regression. Factors will include the presence or absence of low T_3 syndrome, genetic polymorphisms, selenium or iodine insufficiency; cardiac mortality and presence or absence of depressive disorder. For concentrations, we will apply log transformations where appropriate to normalize distributions, make variance more homogeneous, or linearize relationships. Variables will include thyroid axis hormone

concentrations, scores on psychological rating scales. We will examine distributional assumptions prior to analyses using descriptive tools described above, and using residuals after fitting models. If examination of residuals shows an inappropriate model, we will refit a more appropriate model.

Missing data: The sporadic missing data which may occur if e.g. an assay fails. We will examine the missing at random assumption by assessing differences between those with and without missing data. The actual amount of missing data will probably be very small.

Multiple Comparisons: In any study there is a large number of hypotheses being tested. We will control our error rates using different multiple comparison procedures, depending on the specifics. Prior to analysis of the data, we will write a statistical analysis plan which will designate a primary analysis, on a primary outcome measure, for each hypothesis. This will be tested using an uncorrected significance level (two-tailed, 0.05). When the multiple comparisons arise from a number of related response variables, we will use Bonferonni's or Sidak's methods, as appropriate. When there is a clear hierarchy of comparisons, we will use step-down or protected methods. In the exploratory analyses we will not correct for multiple comparisons, but report results as hypothesis generating rather than hypothesis testing.

Discussion

A main model of the proposed research is that decreased serum T_3 concentrations after MI is associated with interaction between specific genetic predispositions, evident by specific SNPs responsible for poor tissue conversion of T_4 to T_3 or transport of thyroid hormone to tissues, and environmental factors, such as selenium and iodine deficiency. Low T_3 syndrome caused by MI or stroke or genetic and environmental factors predicting low T_3 syndrome may affect thyroid hormone homeostasis in the heart or brain contributing to worse survival and worse well-being of CVD patients. Impaired well-being of patients may be evident by symptoms of depression, cognitive dysfunction or fatigue resulting in poor health related quality of life. However, there is no information in patients after acute cardiovascular events if manifestation or compensation of low T_3 syndrome is associated with any genetic markers.

This research project is designed to establish gene-environment interactions between CVD and low T_3 syndrome in patients after MI. These interactions will provide a background for further research finding new effective treatments of cardiac disease or their co-morbidities. Such a clinical trial may cover several approaches:

Administration of T_3 and thyroid hormone analogs in compensation of low T_3 syndrome in cardiac patients, and in psychiatric patients. Some preliminary results show potential use of T_3 in patients with heart failure or in patients after MI. It looks that low T_3 syndrome may play a significant role

in the remodeling processes in the heart. Group of Dr. Bunevicius is collaborating with cardiologists from Italy [9, 10, 13] and Greece exploring such possibilities.

TRH and analogs in cardiac patients, in psychiatric patients and in co-morbid patients may also be used to compensate low T_3 syndrome. One the other hand, TRH or analogs may have physiological effects that are not directly related to thyroid axis functioning. Several new indications for the use of TRH or analogs are already covered by patents, e.g. cancer related fatigue.

All the above mentioned studies may have a strong pharmacogenetic component that may be disclosed by the proposed project.

Environmental factors such dietary selenium or iodine deficiency may also impact development of low T_3 syndrome in MI patients. Substitution with selenium or iodine may be needed to manage patients after acute cardiovascular events.

We expect that the project will give important new knowledge in understanding mechanisms by which gene and environmental factors are associated with cardiovascular events, and by which it affects outcomes and well-being of patients. It will also provide pharmacogenetic foundation for new clinical trials evaluating endocrine, cardiovascular and dietary interventions in patients after acute MI.

The study protocol was approved by Kaunas Regional Biomedical Research Ethics Committee (Registration number 29-04-2013, No. BE-2-11).

Acknowledgements

This research was funded by the European Social Fund under the Global Grant measure, Grant VP1-3.1-SˇMM-07-K- 02-060.

The original version of the study protocol has been published in Lithuanian:

Burkauskas, J. *et al.* Gene-environment interactions connecting Low triiodothyronine syndrome and outcomes of cardiovascular disease (GETVASC): study protocol. *Biological Psychiatry and Psychopharmacology* **16**, 66-73 (2014)

References

1. Eurostat, *Health Statistics: Atlas on mortality in the European Union*. 2009 ed. 2011, Luxembourg: Office for Official Publications of the European Communities.
2. Dickens, C., et al., *New onset depression following myocardial infarction predicts cardiac mortality.* Psychosom Med, 2008. **70**(4): p. 450-5.
3. Friedmann, E., et al., *Relationship of depression, anxiety, and social isolation to chronic heart failure outpatient mortality.* Am Heart J, 2006. **152**(5): p. 940.e1-8.
4. Bunevicius, A., et al., *Relationship of fatigue and exercise capacity with emotional and physical state in patients with coronary artery disease admitted for rehabilitation program.* Am Heart J, 2011. **162**(2): p. 310-6.
5. Fini, A. and D. de Almeida Lopes Monteiro da Cruz, *Characteristics of fatigue in heart failure patients: a literature review.* Rev Lat Am Enfermagem, 2009. **17**(4): p. 557-65.
6. Burkauskas, J., J. Brozaitiene, and R. Bunevicius, *P.4.031 Effects of mental distress on cognitive functioning in patients admitted for cardiac rehabilitation after acute coronary events.* European Neuropsychopharmacology, 2013. **23, Supplement 1**(0): p. S92-S93.
7. Pedersen, S.S. and J. Denollet, *Type D personality, cardiac events, and impaired quality of life: a review.* Eur J Cardiovasc Prev Rehabil, 2003. **10**(4): p. 241-8.
8. Brink, E., et al., *Health-related quality of life in women and men one year after acute myocardial infarction.* Qual Life Res, 2005. **14**(3): p. 749-57.
9. Iervasi, G., et al., *Low-T3 syndrome: a strong prognostic predictor of death in patients with heart disease.* Circulation, 2003. **107**(5): p. 708-13.
10. Bunevičius, R., *Low Triiodothyronine Syndrome and Depression in Patients with Chronic Heart Failure*, in *Thyroid and Heart Failure*, G. Iervasi and A. Pingitore, Editors. 2009, Springer Milan. p. 203-212.
11. Bunevicius, A., et al., *Low triiodothyronine syndrome as a predictor of poor outcomes in patients undergoing brain tumor surgery: a pilot study: clinical article.* J Neurosurg, 2013. **118**(6): p. 1279-87.
12. Pingitore, A., et al., *Acute myocardial infarction and thyroid function: new pathophysiological and therapeutic perspectives.* Ann Med, 2012. **44**(8): p. 745-57.
13. Pingitore, A., et al., *Acute effects of triiodothyronine (T3) replacement therapy in patients with chronic heart failure and low-T3 syndrome: a randomized, placebo-controlled study.* J Clin Endocrinol Metab, 2008. **93**(4): p. 1351-8.
14. van der Deure, W.M., et al., *Polymorphisms in the brain-specific thyroid hormone transporter OATP1C1 are associated with fatigue and depression in hypothyroid patients.* Clin Endocrinol (Oxf), 2008. **69**(5): p. 804-11.
15. Panicker, V., et al., *Common variation in the DIO2 gene predicts baseline psychological well-being and response to combination thyroxine plus triiodothyronine therapy in hypothyroid patients.* J Clin Endocrinol Metab, 2009. **94**(5): p. 1623-9.
16. Schomburg, L., *Selenium, selenoproteins and the thyroid gland: interactions in health and disease.* Nat Rev Endocrinol, 2012. **8**(3): p. 160-71.
17. Frasure-Smith, N. and F. Lesperance, *Depression and anxiety as predictors of 2-year cardiac events in patients with stable coronary artery disease.* Arch Gen Psychiatry, 2008. **65**(1): p. 62-71.
18. Lesperance, F., et al., *Five-year risk of cardiac mortality in relation to initial severity and one-year changes in depression symptoms after myocardial infarction.* Circulation, 2002. **105**(9): p. 1049-53.
19. Lett, H.S., et al., *Depression as a risk factor for coronary artery disease: evidence, mechanisms, and treatment.* Psychosom Med, 2004. **66**(3): p. 305-15.
20. Rugulies, R., *Depression as a predictor for coronary heart disease. a review and meta-analysis.* Am J Prev Med, 2002. **23**(1): p. 51-61.

21. Pragodpol, P. and C. Ryan, *Critical review of factors predicting health-related quality of life in newly diagnosed coronary artery disease patients.* J Cardiovasc Nurs, 2013. **28**(3): p. 277-84.
22. de Jong, F.J., et al., *The association of polymorphisms in the type 1 and 2 deiodinase genes with circulating thyroid hormone parameters and atrophy of the medial temporal lobe.* J Clin Endocrinol Metab, 2007. **92**(2): p. 636-40.
23. Sheehan, D.V., et al., *The Mini-International Neuropsychiatric Interview (M.I.N.I.): the development and validation of a structured diagnostic psychiatric interview for DSM-IV and ICD-10.* J Clin Psychiatry, 1998. **59 Suppl 20**: p. 22-33;quiz 34-57.
24. Janavs, J., *Struktūriniai diagnostiniai interviu ir MINI.* Biologinė psichiatrija ir psichofarmakologija, 2002. **4**(1): p. 50-51.
25. Folstein, M.F., S.E. Folstein, and P.R. McHugh, *"Mini-mental state". A practical method for grading the cognitive state of patients for the clinician.* J Psychiatr Res, 1975. **12**(3): p. 189-98.
26. Bunevicius, R., *Protinės būklės mini tyrimas.* Biologinė psichiatrija ir psichofarmakologija, 2000. **2**(1): p. 13.
27. Zigmond, A.S. and R.P. Snaith, *The hospital anxiety and depression scale.* Acta Psychiatr Scand, 1983. **67**(6): p. 361-70.
28. Bunevicius, A., et al., *Screening for depression and anxiety disorders in primary care patients.* Depress Anxiety, 2007. **24**(7): p. 455-60.
29. Bunevicius, A., et al., *Specific fatigue-related items in self-rating depression scales do not bias an association between depression and fatigue in patients with coronary artery disease.* Gen Hosp Psychiatry, 2011. **33**(5): p. 527-9.
30. Burkauskas, J. and R. Bunevicius, *HAD skalė: depresijos ir nerimo sutrikimų vertinimo instrumentas.* Biologinė psichiatrija ir psichofarmakologija, 2013. **15**(2): p. 59-61.
31. Beck, A.T., et al., *Comparison of Beck Depression Inventories -IA and -II in psychiatric outpatients.* J Pers Assess, 1996. **67**(3): p. 588-97.
32. Gosling, S.D., P.J. Rentfrow, and W.B. Swann Jr, *A very brief measure of the Big-Five personality domains.* Journal of Research in Personality, 2003. **37**(6): p. 504-528.
33. Rector, T.S., S.H. Kubo, and J.N. Cohn, *Patients' self-assessment of their congestive heart failure. Part 2: content, reliability and validity of a new measure, the Minnesota Living with Heart Failure Questionnaire.* Heart failure, 1987. **3**(5): p. 198-209.
34. Ware, J.E., et al., *SF-36 health survey : manual and interpretation guide*. 1993, Boston: Health Institute, New England Medical Center.
35. Brooks, R.G., R. Rabin, and F. De Charro, *The measurement and valuation of health status using EQ-5D : a European perspective : evidence from the EuroQol BIOMED Research Programme*. 2003, Dordrchet; Boston: Kluwer Academic Pub.
36. Stankus, A., *Daugiamatis nuovargio inventorius.* Biologinė psichiatrija ir psichofarmakologija, 2007. **9**(2): p. 86-7.
37. Spitzer, R.L., K. Kroenke, and J.B. Williams, *Validation and utility of a self-report version of PRIME-MD: the PHQ primary care study. Primary Care Evaluation of Mental Disorders. Patient Health Questionnaire.* Jama, 1999. **282**(18): p. 1737-44.
38. Buysse, D.J., et al., *The Pittsburgh Sleep Quality Index: a new instrument for psychiatric practice and research.* Psychiatry Res, 1989. **28**(2): p. 193-213.
